# Leak Detection in Water Pipes Based on Maximum Entropy Version of Least Square Twin K-Class Support Vector Machine

**DOI:** 10.3390/e23101247

**Published:** 2021-09-25

**Authors:** Mingyang Liu, Jin Yang, Wei Zheng

**Affiliations:** Key Laboratory of Optoelectronic Technology & Systems (Ministry of Education), Department of Optoelectronic Engineering, Chongqing University, Chongqing 400044, China; 20160802053@cqu.edu.cn (M.L.); zw3475@cqu.edu.cn (W.Z.)

**Keywords:** leak detection, outliers, LST-KSVC, maximum entropy, MLT-KSVC

## Abstract

Numerous novel improved support vector machine (SVM) methods are used in leak detection of water pipelines at present. The least square twin K-class support vector machine (LST-KSVC) is a novel simple and fast multi-classification method. However, LST-KSVC has a non-negligible drawback that it assigns the same classification weights to leak samples, including outliers that affect classification, these outliers are often situated away from the main leak samples. To overcome this shortcoming, the maximum entropy (MaxEnt) version of the LST-KSVC is proposed in this paper, called the MLT-KSVC algorithm. In this classification approach, classification weights of leak samples are calculated based on the MaxEnt model. Different sample points are assigned different weights: large weights are assigned to primary leak samples and outliers are assigned small weights, hence the outliers can be ignored in the classification process. Leak recognition experiments prove that the proposed MLT-KSVC algorithm can reduce the impact of outliers on the classification process and avoid the misclassification color block drawback in linear LST-KSVC. MLT-KSVC is more accurate compared with LST-KSVC, TwinSVC, TwinKSVC, and classic Multi-SVM.

## 1. Introduction

Water supply pipelines are important infrastructure in cities, and maintaining the stable operation of water supply pipelines has significant economic, sanitary, and environmental worth. Therefore, the real-time monitoring of pipeline operation status and detection of suspected leak risks are significant for maintaining the safe operation of pipe network, avoiding water resource waste, and realizing sustainable production [1].

As a vital technology in the machine learning field, the support vector machine (SVM) [2] and its improved versions are widely utilized in pipeline leak detection and localization. To achieve greater efficiency in leak detection in water pipes, a novel improved multi-class SVM algorithm is herein proposed, called maximum entropy [3] (MaxEnt) version of LST-KSVC [4] (MLT-KSVC). This paper is organized as follows. Various leak detection and location methods proposed in recent years are summarized in Section 2. The theoretical explanation of LST-KSVC and MLT-KSVC is presented in Section 3. Experimental setup and data processing are presented in Section 4. Finally, the conclusions are offered in Section 5.

## 2. Related Work

In recent years, several leak detection and location methods based on artificial intelligence algorithms have been proposed. This paper divides these technologies into two categories: (1) leak recognition or detection method based on machine learning algorithms [5,6,7,8] and (2) leak recognition or detection method based on deep learning algorithms [9,10,11,12]. These two methods collect leak acoustic signal, pressure signal, flow signal, or transient water hammer wave signal of the water pipes to build leak dataset.

In the leak detection system based on machine learning algorithms, Bohorquez [13] presented a methodology that uses artificial neural networks (ANNs) to predict the presence of leak features in a pipeline. This methodology demonstrated the potential of the combined use of both fluid transient pressure waves and ANNs to detect leak features in pipelines. Pérez [14] also proposed an improved ANNs leak diagnosis for fluid transport pipelines. In this methodology, the pressure and flow rate were acquired as original data for ANNs, and the pipe friction factor was used as an input to estimate the leak point. Gong [15] proposed a pipe leak detection method based on acoustic emission (AE) data and neural networks. In this detection method, a leak classifier was built based on a backpropagation neural network after leak feature extraction and analysis from AE signals. Diao [16] combined particle swarm optimization (PSO) algorithm, MaxEnt, and variational mode decomposition (VMD) to remove background noise from leak AE signals. Then, the SVM was employed to complete leak recognition for de-noised leak data. Quy [17] used the spectral portrait method to pre-process pipe leak AE signals. Next, a multi-class SVM classifier was used for leak detection.

In the leak detection system based on deep learning algorithms, Kang [18] combined a one-dimensional convolutional neural network (CNN) and SVM to solve computational and time cost problems in an online pipe leak detection. Cody [19] combined CNN with variational autoencoder to detect small leaks in buried water pipelines. Lang [20] recommended a method to detect small leak apertures in pipelines. The method was composed of wavelet packet analysis (WPA) and deep belief network (DBN) with independent component regression (ICR).

However, these methods fail to remove the interference of noise. When SVM and its improved algorithms are used in pipeline leak monitoring, outliers often disturb the classification processing. Such outliers are distant from the data sample center and they are mainly brought about by interference noise. To overcome this shortcoming, this paper introduces a novel algorithm, the MLT-KSVC algorithm, which is based on the recently presented LST-KSVC algorithm. Unlike the LST-KSVC algorithm, which assigns the same classification weights to all data samples, MLT-KSVC uses the MaxEnt model to build two classification weight matrices for leak samples. In these matrices, outliers are assigned small values, minimizing their negative effect on classification, and effectively solving the problem of outlier interference to the classification algorithm. Some researchers have combined entropy theory with SVMs algorithm. Zhang [21] used sample entropy to extract features from multichannel electroencephalography (EEG) signals; the extracted features were used for the classification of classic SVM. Xie [22] combined optimized variational mode decomposition, permutation entropy, and normalized Spearman correlation coefficient to extract features from ship-radiated noise (S-RN) signals. Then, these features were classified by a multi-class SVM algorithm. However, most of these combinations are not directly aimed at outliers, and their effect on outliers is very limited.

## 3. Theory

### 3.1. Background of LST-KSVC

LST-KSVC is a novel multi-class classification algorithm that uses the "one-versus-one-versus-rest" strategy [23] to evaluate all training samples with ternary output {−1, 0, +1}. In this section, we use $D=\left\{\left(x_{1},y_{1}\right),\left(x_{2},y_{2}\right),\ldots ,\left(x_{m},y_{m}\right)\right\}$ as the training data set. Where $x_{i}$ represents the input sample in the m-dimensional real space $R_{m}$ and $y_{i}\in N^{q}$ is the *q*-class outputs i=1,…,m. In the LST-KSVC classification, the formulas for two non-parallel hyperplanes are as follows:

(1) $$\{\begin{array}{l} w_{1}^{+}x+b_{+}=0\\ w_{2}^{-}x+b_{-}=0 \end{array}$$

where $w_{1}^{+}\;\mathrm{and}\;w_{2}^{-}\in R^{n}$ are the normal matrices of the hyperplane, but $b_{+}$ and $b_{-}\in R$ are two constants. The decision functions of LST-KSVC are obtained by the following two optimization functions:

(2) $$\underset{w_{1}^{+},b_{+},\delta ,\xi ,\eta }{\min}\frac{1}{2}\delta^{T}\delta +\frac{c_{1}}{2}\xi^{T}\xi +\frac{c_{2}}{2}\eta^{T}\eta$$

$$s.t.\{\begin{array}{l} Aw_{1}^{+}+e_{+}b_{+}=\delta \\ -e_{-}-\left(Bw_{1}^{+}+e_{-}b_{+}\right)=\xi \\ e_{0}\left(\varepsilon -1\right)-\left(Cw_{1}^{+}+e_{0}b_{+}\right)=\eta \end{array}$$

and

(3) $$\underset{w_{2}^{-},b_{-},\delta^{*},\xi^{*},\eta^{*}}{\min}\frac{1}{2}\xi^{*T}\xi^{*}+\frac{c_{3}}{2}\delta^{*T}\delta^{*}+\frac{c_{4}}{2}\eta^{*T}\eta^{*}$$

$$s.t.\{\begin{array}{l} Bw_{2}^{-}+e_{-}b_{-}=\xi^{*}\\ e_{+}-\left(Aw_{2}^{-}+e_{+}b_{-}\right)=\delta^{*}\\ e_{0}\left(1-\varepsilon \right)-\left(Cw_{2}^{-}+e_{0}b_{-}\right)=\eta^{*} \end{array}$$

In Equations (2) and (3), δ and $\delta^{*}$, ξ and $\xi^{*}$, and η and $\eta^{*}$ belong to the *l*_1_-dimensional real space, *l*_2_-dimensional real space, and *l*_3_-dimensional real space, respectively. $A,B,C\in R^{l_{i}\times n}\left(i=1,2,3\right)$, $c_{i}\;\left(i=1,\ldots ,4\right)$, and ε are positive real factors. *e*_1_ and *e*_2_, *e*_0_ are vectors of appropriate dimensions. The final classification decision functions of LST-KSVC in the linear case are determined as:

(4) $$f\left(x\right)=\{\begin{array}{l} +1,\;\;\mathrm{if}\;x^{T}w_{1}^{+}+b_{+}>-1+\varepsilon \;\\ -1,\;\;\mathrm{if}\;x^{T}w_{2}^{-}+b_{-}<1-\varepsilon \\ 0,\;\;\;\;\;otherwise\;\; \end{array}$$

The decision functions of LST-KSVC in the nonlinear case are determined as:

(5) $$f\left(x\right)=\{\begin{array}{l} +1,\;\;\mathrm{if}\;k\left(x^{T},D^{T}\right)w_{1}^{+}+b_{+}>-1+\varepsilon \;\\ -1,\;\;\mathrm{if}\;k\left(x^{T},D^{T}\right)w_{2}^{-}+b_{-}<1-\varepsilon \\ 0,\;\;\;\;\;otherwise\;\; \end{array}\;$$

### 3.2. MLT-KSVC

#### 3.2.1. Background of MaxEnt Model

The principle of MaxEnt is to find the largest entropy model from the probability model set that satisfies the known constraint conditions. Given a data set ${\left\{x_{i},\;y_{i}\right\}}_{i=1}^{N}$, the feature function of the data set is $f_{i}\left(x,y\right),\;i=1,2,\ldots n$, and the constraints of the MaxEnt model are obtained according to the empirical distribution condition:

(6) $$\underset{x,y}{\sum }\tilde{P}\left(x\right)p\left(y|x\right)f\left(x,y\right)=\underset{x,y}{\sum }\tilde{P}\left(x,y\right)f\left(x,y\right)$$

Assume that all sets C satisfying the constraints are:

(7) $$C=E_{P}\left(f_{i}\right)=E_{\tilde{P}}\left(f_{i}\right),i=1,2,\ldots ,n$$

The conditional entropy is defined on the conditional probability distribution P(Y|X) is:

(8) $$H\left(P\right)=-\underset{x,y}{\sum }P\left(y,x\right)\mathrm{lg}P\left(y|x\right)=-\underset{x,y}{\sum }\tilde{P}\left(x\right)P(y|x)\mathrm{lg}P(y|x)$$

The goal is to find the corresponding P(y|x) when H(P) is the largest. Here, a minus sign is added to H(P) to find the extreme minimum value. To make −H(P) a convex function, it is convenient to use the convex optimization method to find the extreme value. Therefore, the loss function for MaxEnt is:

(9) $$P^{*}=arg\underset{P\in C}{\max}H\left(P\right)$$

The objective function of the MaxEnt model is:

(10) $$\underset{P\in C}{\min}\underset{x,y}{\sum }\tilde{P}\left(x\right)P(y|x)\mathrm{lg}P(y|x)$$

(11) $$s.t.\;E_{p}\left(f_{i}\right)=E_{\tilde{p}}\left(f_{i}\right),\;\underset{y}{\sum }P\left(y|x\right)=1$$

The objective function of the MaxEnt model is an optimization problem with constraints. Based on the principle of Lagrangian duality, this problem can be transformed into an unconstrained optimization problem. First, we introduce a series of Lagrangian multipliers $\omega_{0},\;\omega_{1},\ldots ,\;\omega_{n}$, and define the Lagrangian function L(P,ω) corresponding to this objective function:

(12) $$L\left(P,\omega \right)=-H\left(P\right)+\omega_{0}\left[1-\underset{y}{\sum }P\left(y|x\right)\right]+\sum_{i=1}^{n}\omega_{i}\left(E_{\tilde{P}}\left(f_{i}\right)-E_{P}\left(f_{i}\right)\right)$$

Next, the optimization problem is transformed into $\underset{P\in C}{\min}L\left(P,\omega \right)$, where the Lagrangian function L(P,ω) must meet the constraints to obtain the extreme minimum value. After satisfying the constraints, L(P,ω)=maxL(P,ω) is obtained, and then the optimization problem is transformed into an extreme minimum-maximum solution problem that is convenient for Lagrangian dual calculation:

(13) $$\underset{P\in C}{\min}\;\underset{\omega }{\max}L\left(P,\omega \right)$$

Since L(P,ω) is a convex function with respect to P, according to the Lagrangian duality, the extreme minimum-maximum problem of L(P,ω) is equivalent to the extreme maximum-minimum problem:

(14) $$\underset{P\in C}{\min}\;\underset{\omega }{\max}L\left(P,\omega \right)=\underset{\omega }{\max}\;\underset{P\in C}{\min}L\left(P,\omega \right)$$

Next, we find the extreme minimal problem $\underset{P\in C}{\min}L\left(P,\omega \right)$ of $\underset{\omega }{\max}\;\underset{P\in C}{\min}L\left(P,\omega \right)$, and $\underset{P\in C}{\min}L\left(P,\omega \right)$ is solved to obtain the function of ω, denoted as Ψ(ω):

(15) $$\Psi \left(\omega \right)=\underset{P\in C}{\min}L\left(P,\omega \right)=L\left(P_{\omega },\omega \right)$$

The solution $P_{\omega }$ of the above formula is:

(16) $$P_{\omega }=arg\underset{P\in C}{\min}L\left(P,\omega \right)=P_{\omega }\left(y|x\right)={\left[Z_{\omega }\left(x\right)\right]}^{-1}\exp\left(\overset{n}{\underset{i=1}{\sum }}\omega_{i}f_{i}\left(x,y\right)\right)$$

(17) $$Z_{\omega }\left(x\right)=\underset{y}{\sum }exp\left[\overset{n}{\underset{i=1}{\sum }}\omega_{i}f_{i}\left(x,y\right)\right]$$

In Equation (17), $f_{i}\left(x,y\right)$ represents the feature function and $\omega_{i}$ is the weight value of the feature function, and thus $P_{\omega }\left(y|x\right)$ is the MaxEnt model. The minimization problem is solved to obtain the weight value function of ω, and the solved optimal solution is recorded as $\omega^{*}$:

(18) $$\omega^{*}=arg\underset{\omega }{\max}\left[\underset{x,y}{\sum }\tilde{P}\left(x,y\right)\sum_{i=1}^{n}\omega_{i}f_{i}\left(x,y\right)+\underset{x}{\sum }\tilde{P}\left(x\right)\mathrm{lg}Z_{\omega }\left(x\right)\right]$$

A series of obtained weight values $\omega_{i}^{*}$ are filled into the matrix $W_{1+}$. Then, $\omega_{i}^{*}$ are reversed to obtain $-\omega_{i}^{*}$ and filled into matrix $W_{2-}$. Because outliers are distant from the data center, their probability (P) value belonging to the data set is the lowest among all sample points. Hence, the weight value corresponding to outliers is much smaller than other normal sample points.

#### 3.2.2. Linear MLT-KSVC

Similar to LST-KSVC, MLT-KSVC also has two hyperplanes. The two hyperplanes in linear MLT-KSVC are defined as follows:

(19) $$\{\begin{array}{l} w_{1}^{+}x+b_{+}=0\\ w_{2}^{-}x+b_{-}=0 \end{array}$$

where $w_{1}^{+}$ and $w_{2}^{-}$ are normal matrices of the hyperplane, $w_{1}^{+},w_{2}^{-}\in R^{n}$, $b_{+}$ and $b_{-}$ belong to R real number space. Next, $W_{1+}$ and $W_{2-}$ matrices are introduced into the objective functions of MLT-KSVC. This operation makes MLT-KSVC avoid the negative impact of outliers to the greatest extent. The obtained objective functions of MLT-KSVC are as follows:

(20) $$\underset{w_{1}^{+},b_{+},\alpha ,\gamma ,\eta }{\min}\frac{1}{2}\alpha^{T}\alpha +\frac{c_{1}}{2}\gamma^{T}\gamma +c_{2}\lambda^{T}\eta$$

$$s.t.\{\begin{array}{l} W_{1+}\left(Aw_{1}^{+}+e_{+}b_{+}\right)=\alpha \\ W_{2-}\left\{-e_{-}-\left(Bw_{1}^{+}+e_{-}b_{+}\right)\right\}=\gamma \\ e_{0}\left(\gamma -1\right)-\left(Cw_{1}^{+}+e_{0}b_{+}\right)=\eta \end{array}$$

and

(21) $$\underset{w_{2}^{-},b_{-},\alpha^{*},\gamma^{*},\eta^{*}}{\min}\frac{1}{2}\gamma^{*T}\gamma^{*}+\frac{c_{3}}{2}\alpha^{*T}\alpha^{*}+c_{4}\lambda^{*T}\eta^{*}$$

$$s.t.\{\begin{array}{l} W_{2-}\left(Bw_{2}^{-}+e_{-}b_{-}\right)=\gamma^{*}\\ W_{1+}\left\{e_{+}-\left(Aw_{2}^{-}+e_{+}b_{-}\right)\right\}=\alpha^{*}\\ e_{0}\left(1-\gamma \right)-\left(Cw_{2}^{-}+e_{0}b_{-}\right)=\eta^{*} \end{array}$$

where $c_{i}\;\left(i=1,\ldots ,4\right)$ is positive real number factor, $W_{1+}$ and $W_{2-}$ are obtained from the MaxEnt model, α and $\alpha^{*}$ are two vectors belonging to the *l*_1_-dimensional real number space, γ and $\gamma^{*}$ are two vectors belonging to the *l*_2_-dimensional real number space, and η and $\eta^{*}$ are two vectors belonging to the *l*_3_-dimensional real number space. The matrices A, B, and C all belong to the real number space $R^{l_{i}\times n}\left(i=1,2,3\right)$, *e*_0_ and *e*_1_, *e*_2_ are three adjustment vectors. λ and $\lambda^{*}$ also belong to the *l*_3_-dimensional real number space. They are calculated by the least-squares linear loss function and can be used to avoid the local convergence phenomenon of the objective function. Next, constraint conditions in Equations (20) and (21) are substitute into the objective functions so that the objective functions are optimized under the constraint conditions. The new objective functions obtained are as follows:

(22) $$\underset{w_{1}^{+},b_{+},\alpha ,\gamma ,\eta }{\min}\frac{1}{2}\|W_{1+}{\left(Aw_{1}^{+}+e_{+}b_{+}\right)\|}^{2}+\frac{c_{1}}{2}\|W_{2-}{\left\{-e_{-}-\left(Bw_{1}^{+}+e_{-}b_{+}\right)\right\}\|}^{2}\;+c_{2}\lambda^{T}\left\{e_{0}\left(\gamma -1\right)-\left(Cw_{1}^{+}+e_{0}b_{+}\right)\right\}$$

and

(23) $$\underset{w_{2}^{-},b_{-},\alpha^{*},\gamma^{*},\eta^{*}}{\min}\frac{1}{2}\|W_{2-}{\left(Bw_{2}^{-}+e_{-}b_{-}\right)\|}^{2}+\frac{c_{3}}{2}\|W_{1+}{\left\{e_{+}-\left(Aw_{2}^{-}+e_{+}b_{-}\right)\right\}\|}^{2}\;+c_{4}\lambda^{*T}\left\{e_{0}\left(1-\gamma \right)-\left(Cw_{2}^{-}+e_{0}b_{-}\right)\right\}$$

It can be seen that Equations (22) and (23) are two minimization problems. Partial derivatives of $w_{1}^{+}$, $b_{+}$ and $w_{2}^{-}$, $b_{-}$ are respectively determined from Equations (22) and (23), and then all partial derivatives are equal to zero:

(24) $$\begin{array}{l} W_{1+}A\left(W_{1+}Aw_{1}^{+}+W_{1+}e_{+}b_{+}\right)+c_{1}W_{2-}B\left(W_{1+}Bw_{1}^{+}+W_{1+}e_{-}b_{+}+W_{1+}e_{-}\right)-c_{2}\lambda^{*T}C=0\\ W_{1+}e_{+}\left(W_{1+}Aw_{1}^{+}+W_{1+}e_{+}b_{+}\right)+c_{1}W_{2-}e_{-}\left(W_{1+}Bw_{1}^{+}-W_{1+}e_{-}b_{+}-W_{1+}e_{-}\right)-c_{2}\lambda^{*T}e_{0}=0 \end{array}$$

(25) $$\begin{array}{l} W_{2-}B\left(W_{2-}Bw_{2}^{-}+W_{2-}e_{-}b_{-}\right)+c_{3}W_{1+}A\left(W_{1+}Aw_{2}^{-}-W_{1+}e_{+}b_{-}+W_{1+}e_{+}\right)-c_{4}\lambda^{*T}C=0\\ W_{2-}e_{-}\left(W_{2-}Bw_{1}^{+}+W_{2-}e_{-}b_{-}\right)+c_{3}W_{1+}e_{+}\left(W_{1+}Aw_{2}^{-}+W_{1+}e_{+}b_{-}-W_{1+}e_{+}\right)-c_{4}\lambda^{*T}e_{0}=0 \end{array}$$

Subsequently, Equations (24) and (25) are organized into the matrix forms (Equations (26) and (27)).

(26) $$\{\begin{array}{l} c_{2}\lambda^{*T}\left[\begin{array}{l} C\\ e_{0} \end{array}\right]{\left\{M+c_{1}N\right\}}^{-1}=\left[\begin{array}{l} w_{1}^{+}\\ b_{+} \end{array}\right]\\ M=\left[\begin{array}{ll} W_{1+}AW_{1+}A & -W_{1+}AW_{1+}e_{+}\\ -W_{1+}e_{+}W_{1+}A & W_{1+}e_{+}W_{1+}e_{+} \end{array}\right]\\ N=\left[\begin{array}{ll} W_{2-}BW_{1+}B & -W_{1+}BW_{1}^{-}e_{-}\\ -W_{1+}e_{-}W_{2-}B & W_{2-}e_{-}W_{1+}e_{-} \end{array}\right] \end{array}$$

(27) $$\{\begin{array}{l} c_{4}\lambda^{*T}\left[\begin{array}{l} C\\ e_{0} \end{array}\right]{\left\{M+c_{3}N\right\}}^{-1}=\left[\begin{array}{l} w_{2}^{-}\\ b_{-} \end{array}\right]\\ M=\left[\begin{array}{ll} W_{2-}BW_{2-}B & -W_{2-}BW_{2-}e_{-}\\ -W_{1+}e_{+}W_{1+}A & W_{1+}e_{+}W_{1+}e_{+} \end{array}\right]\\ N=\left[\begin{array}{ll} W_{1+}AW_{1+}A & -W_{1+}AW_{1+}e_{+}\\ -W_{2-}e_{+}W_{1}^{+}A & W_{1+}e_{-}W_{1+}e_{-} \end{array}\right] \end{array}$$

Therefore, the solutions for $w_{1}^{+}$, $b_{+}$ and $w_{2}^{-}$, $b_{-}$ can be solved by Equations (26) and (27). After $w_{1}^{+}$, $b_{+}$ and $w_{2}^{-}$, $b_{-}$ are solved, Equation (19) can be used to construct two linear classification hyperplanes of MLT-KSVC. The proposed linear MLT-KSVC is summarized in Algorithm 1.
**Algorithm 1**: Linear MLT-KSVC(1) Initialize matrices $A,\;B,\;\mathrm{and}\;C\;\in \;R^{l_{i}\times n}\left(i=1,2,3\right)$, ***e***_0_ and ***e***_1_, ***e***_2_.(2) Run the program based on MaxEnt structure to obtain the weight matrices $W_{1+}$, $W_{2-}$.(3) Select the kernel parameter as “linear”, and use the grid search method to optimize the hyperparameter C and penalty factor G.(4) Initialize $w_{1}^{+}$, $b_{+}$ and $w_{2}^{-}$, $b_{-}$, α and $\alpha^{*}$, γ and $\gamma^{*}$, η and $\eta^{*}$, λ and $\lambda^{*}$.(5) For iter ≥ 0:Calculate$c_{2}\lambda^{*T}\left[\begin{array}{l} C\\ e_{0} \end{array}\right]{\left\{M+c_{1}N\right\}}^{-1}=\left[\begin{array}{l} w_{1}^{+}\\ b_{+} \end{array}\right]$ $c_{4}\lambda^{*T}\left[\begin{array}{l} C\\ e_{0} \end{array}\right]{\left\{M+c_{3}N\right\}}^{-1}=\left[\begin{array}{l} w_{2}^{-}\\ b_{-} \end{array}\right]$(6) End for convergence and obtain the optimal solutions: $w_{1}^{+}$, $b_{+}$ and $w_{2}^{-}$, $b_{-}$.

#### 3.2.3. Nonlinear MLT-KSVC

Considering that the distribution of sample points is not regularly linearly separable in real classification, extending the linear classification theory of MLT-KSVC to a nonlinear version is necessary. In nonlinear MLT-KSVC case, two classification hyperplanes are no longer linear functions, they are defined as follows:

(28) $$\{\begin{array}{l} Kw_{1}^{+}\left(x^{T},D^{T}\right)+b_{+}=0\\ Kw_{2}^{-}\left(x^{T},D^{T}\right)+b_{-}=0 \end{array}$$

where K(·) is an arbitrary kernel function [24]. It maps the complex linear inseparable problem to a high-dimensional space, transforming the linear inseparable problem into a linear separable problem. Similar to the linear MLT-KSVC, after the classification hyperplanes are obtained, the objective functions for solving $w_{1}^{+}$, $b_{+}$ and $w_{2}^{-}$, $b_{-}$ should be defined. The objective functions of the nonlinear MLT-KSVC are defined as follows:

(29) $$\underset{w_{1}^{+},b_{+},\alpha ,\gamma ,\eta }{\min}\frac{1}{2}\alpha^{T}\alpha +\frac{c_{1}}{2}\gamma^{T}\gamma +c_{2}\lambda^{T}\eta$$

$$s.t.\{\begin{array}{l} W_{1+}\left(Kw_{1}^{+}\left(A,D^{T}\right)+e_{+}b_{+}\right)=\alpha \\ W_{2-}\left\{-e_{-}-\left(Kw_{1}^{+}\left(B,D^{T}\right)+e_{-}b_{+}\right)\right\}=\gamma \\ e_{0}\left(\gamma -1\right)-\left(Kw_{1}^{+}\left(C,D^{T}\right)+e_{0}b_{+}\right)=\eta \end{array}$$

and

(30) $$\underset{w_{2}^{-},b_{-},\alpha^{*},\gamma^{*},\eta^{*}}{\min}\frac{1}{2}\gamma^{*T}\gamma^{*}+\frac{c_{3}}{2}\alpha^{*T}\alpha^{*}+c_{4}\lambda^{*T}\eta^{*}$$

$$s.t.\{\begin{array}{l} W_{2-}\left(Kw_{2}^{-}\left(B,D^{T}\right)+e_{-}b_{-}\right)=\gamma^{*}\\ W_{1+}\left\{e_{+}-\left(Kw_{2}^{-}\left(A,D^{T}\right)+e_{+}b_{-}\right)\right\}=\alpha^{*}\\ e_{0}\left(1-\gamma \right)-\left(Kw_{2}^{-}\left(C,D^{T}\right)+e_{0}b_{-}\right)=\eta^{*} \end{array}$$

In the constraints of the objective functions Equation (29) and Equation (30), classification weight matrices $W_{1+}$ and $W_{2-}$ are considered, which implies that the interference of outliers can also be reduced in the nonlinear MLT-KSVC case. The constraints are substituted into objective functions of Equation (29) and Equation (30), and the following formulas obtained:

(31) $$\underset{w_{1}^{+},b_{+},\alpha ,\gamma ,\eta }{\min}\frac{1}{2}\|W_{1+}{\left(Kw_{1}^{+}\left(A,D^{T}\right)+e_{+}b_{+}\right)\|}^{2}\;\;+\frac{c_{1}}{2}\|W_{2-}{\left\{-e_{-}-\left(Kw_{1}^{+}\left(B,D^{T}\right)+e_{-}b_{+}\right)\right\}\|}^{2}\;\;+c_{2}\lambda^{T}\left\{e_{0}\left(\gamma -1\right)-\left(Kw_{1}^{+}\left(C,D^{T}\right)+e_{0}b_{+}\right)\right\}$$

(32) $$\underset{w_{2}^{-},b_{-},\alpha^{*},\gamma^{*},\eta^{*}}{\min}\frac{1}{2}{\left\|W_{2-}\left(Kw_{2}^{-}\left(B,D^{T}\right)+e_{-}b_{-}\right)\right\|}^{2}\;\;+\frac{c_{3}}{2}\|W_{1+}{\left\{e_{+}-\left(Kw_{2}^{-}\left(A,D^{T}\right)+e_{+}b_{-}\right)\right\}\|}^{2}\;\;+c_{4}\lambda^{*T}\left\{e_{0}\left(1-\gamma \right)-\left(Kw_{2}^{-}\left(C,D^{T}\right)+e_{0}b_{-}\right)\right\}$$

Partial derivatives with respect to $w_{1}^{+}$, $b_{+}$ and $w_{2}^{-}$, $b_{-}$ are solved, and then partial derivative equations are transformed in matrix form:

(33) $$\{\begin{array}{l} c_{2}\lambda^{*T}\left[\begin{array}{l} C\\ e_{0} \end{array}\right]{\left\{U+c_{1}V\right\}}^{-1}=\left[\begin{array}{l} w_{1}^{+}\\ b_{+} \end{array}\right]\\ U=\left[\begin{array}{ll} {\left(W_{1+}K\left(A,D^{T}\right)\right)}^{2} & W_{1+}^{2}K\left(A,D^{T}\right)e_{+}\\ W_{1+}^{2}e_{+}K\left(A,D^{T}\right) & {\left(W_{1+}e_{+}\right)}^{2} \end{array}\right]\\ V=\left[\begin{array}{ll} W_{1+}^{2}W_{2-}{\left(K\left(B,D^{T}\right)\right)}^{2} & W_{1+}^{2}K\left(B,D^{T}\right)e_{-}\\ W_{1+}e_{-}W_{2-}K\left(B,D^{T}\right) & W_{2-}W_{1+}e_{-}^{2} \end{array}\right] \end{array}$$

(34) $$\{\begin{array}{l} c_{4}\lambda^{*T}\left[\begin{array}{l} C\\ e_{0} \end{array}\right]{\left\{U+c_{3}V\right\}}^{-1}=\left[\begin{array}{l} w_{2}^{-}\\ b_{-} \end{array}\right]\\ U=\left[\begin{array}{ll} {\left(W_{2-}K\left(B,D^{T}\right)\right)}^{2} & W_{2-}^{2}K\left(B,D^{T}\right)e_{-}\\ W_{1+}^{2}e_{+}K\left(A,D^{T}\right) & {\left(W_{1+}e_{+}\right)}^{2} \end{array}\right]\\ V=\left[\begin{array}{ll} {\left(W_{1+}K\left(A,D^{T}\right)\right)}^{2} & W_{1+}^{2}e_{+}K\left(A,D^{T}\right)\\ W_{2-}W_{1+}e_{+}K\left(A,D^{T}\right) & {\left(W_{1+}e_{-}\right)}^{2} \end{array}\right] \end{array}$$

After obtaining the solutions of $w_{1}^{+}$, $b_{+}$ and $w_{2}^{-}$, $b_{-}$, the nonlinear MLT-KSVC classification hyperplanes are established according to Equation (28). The proposed nonlinear MLT-KSVC is summarized in Algorithm 2.
**Algorithm 2:** Nonlinear MLT-KSVC(1) Initialize matrices $A,\;B,\;\mathrm{and}\;C\;\in \;R^{l_{i}\times n}\left(i=1,2,3\right)$, ***e***_0_ and ***e***_1_, ***e***_2_.(2) Run the program based on MaxEnt structure to obtain the weight matrices $W_{1+}$, $W_{2-}$.(3) Select the kernel parameter as “RBF” or “Gaussian”, and use the grid search method to optimize the hyperparameter *C* and penalty factor *G*.(4) Initialize $w_{1}^{+}$, $b_{+}$ and $w_{2}^{-}$, $b_{-}$ under the selected nonlinear kernel function. (5) Initialize α and $\alpha^{*}$, γ and $\gamma^{*}$, η and $\eta^{*}$, λ and $\lambda^{*}$.(6) For iter ≥ 0:Calculate$c_{2}\lambda^{*T}\left[\begin{array}{l} C\\ e_{0} \end{array}\right]{\left\{U+c_{1}V\right\}}^{-1}=\left[\begin{array}{l} w_{1}^{+}\\ b_{+} \end{array}\right]$ $c_{4}\lambda^{*T}\left[\begin{array}{l} C\\ e_{0} \end{array}\right]{\left\{U+c_{3}V\right\}}^{-1}=\left[\begin{array}{l} w_{2}^{-}\\ b_{-} \end{array}\right]$(7) End for convergence and obtain the optimal solutions: $w_{1}^{+}$, $b_{+}$ and $w_{2}^{-}$, $b_{-}$.

### 3.3. Multi-Classification Rule of MLT-KSVC

MLT-KSVC is an improved algorithm of LST-KSVC. As described in Section 3.1, LST-KSVC is a classification algorithm based on the "one-versus-one-versus-rest" strategy. Therefore, MLT-KSVC is also a classification algorithm based on "one-versus-one-versus-rest" strategy. In the "one-versus-one-versus-rest" strategy, the classification algorithm outputs three labels {+1, 0, −1}. When the classification number is *q* (*q* > 2), *q*(*q* − 1)/2 MLT-KSVC sub-classifiers are required to complete the classification. This classification process is a voting process. In vote classification, MLT-KSVC labels “+1” to *i*-th class samples, “−1” to *j*-th class samples, and “0” to all remaining classes, respectively, where i,j∈{1,2,…,q}. Then, the hyperplane parameters $w_{1}^{+}$, $b_{+}$ and $w_{2}^{-}$, $b_{-}$ of the (*i*, *j*)th sub-classifier are obtained from Equations (26), (27), (33) and (34). In linear MLT-KSVC case, classification labels are determined using the following function:

(35) $$f\left(x\right)=\{\begin{array}{l} +1,\;\;\mathrm{if}\;x^{T}w_{1}^{+}+b_{+}>-1+\varepsilon \;\\ -1,\;\;\mathrm{if}\;x^{T}w_{2}^{-}+b_{-}<1-\varepsilon \\ 0,\;\;\;\;\;otherwise\;\; \end{array}$$

In nonlinear MLT-KSVC case, the corresponding decision function is:

(36) $$f\left(x\right)=\{\begin{array}{l} +1,\;\;\mathrm{if}\;k\left(x^{T},D^{T}\right)w_{1}^{+}+b_{+}>-1+\varepsilon \;\\ -1,\;\;\mathrm{if}\;k\left(x^{T},D^{T}\right)w_{2}^{-}+b_{-}<1-\varepsilon \\ 0,\;\;\;\;\;otherwise\;\; \end{array}$$

Finally, after *q*(*q* − 1)/2 sub-classifiers, the test samples are classified as the label with the most votes.

## 4. MLT-KSVC-Based Leak Detection

### 4.1. Overview of the Recommended Leak Detection Procedure

**Figure 1:**
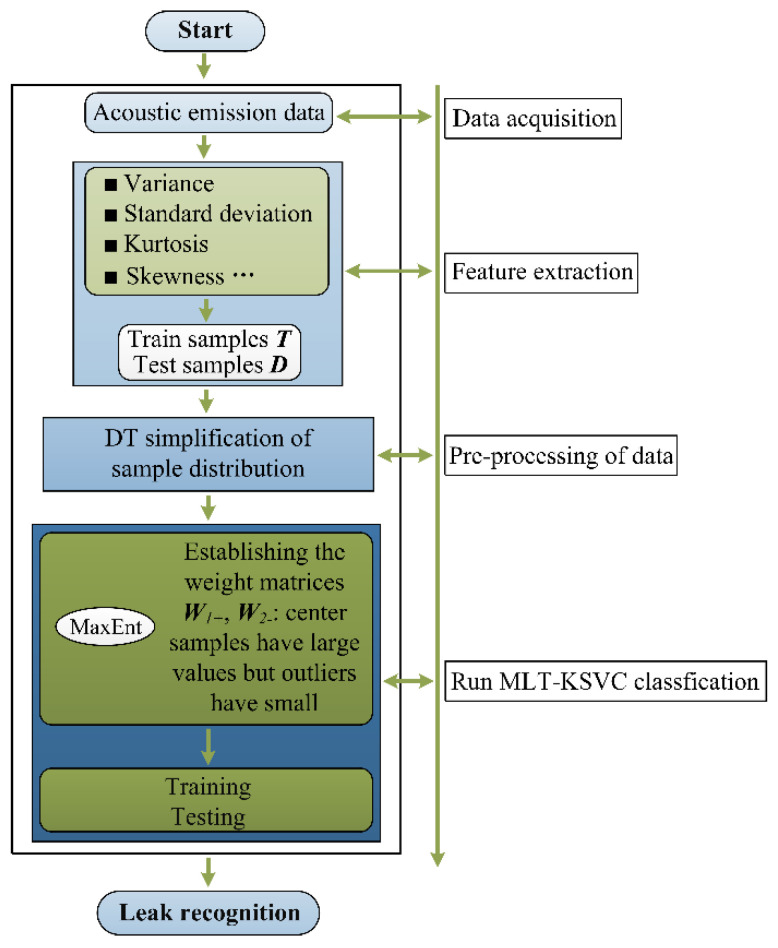
A schematic diagram of pipeline leakage test platform.

In this experimental section, the proposed MLT-KSVC algorithm is used for water supply pipe leak identification. A schematic of the experiment is shown in Figure 1. The experiment includes four steps as follows.

Step 1: Piezoelectric (PZT) acoustic sensor was used to acquire the vibro-acoustic emission (VAE) data on the pipe, which VAE data was used as the data source for the next feature extraction.

Step 2: Eight methods, including standard deviation, kurtosis, variance, RMS, margin, mean, waveform factor, and peak factor, were used to extract feature values from the VAE data source. These feature values have been proven by previous studies [25,26] that they can indicate the features of different leak severity. Then, the extracted features constituted the leak sample data.

Step 3: The extracted feature values were simplified by the Delaunay triangulation (*DT*) algorithm [27]. The simplification process was to remove redundant points in the sample center and retain the mainframe of the samples.

Step 4: According to the distribution characteristics of the sample points, MaxEnt was used to establish classification weight matrices $W_{1+}$ and $W_{2-}$, and then the samples were classified by the proposed algorithm, and finally leak detection was completed.

### 4.2. Acquisition of VAE Data

**Figure 2:**
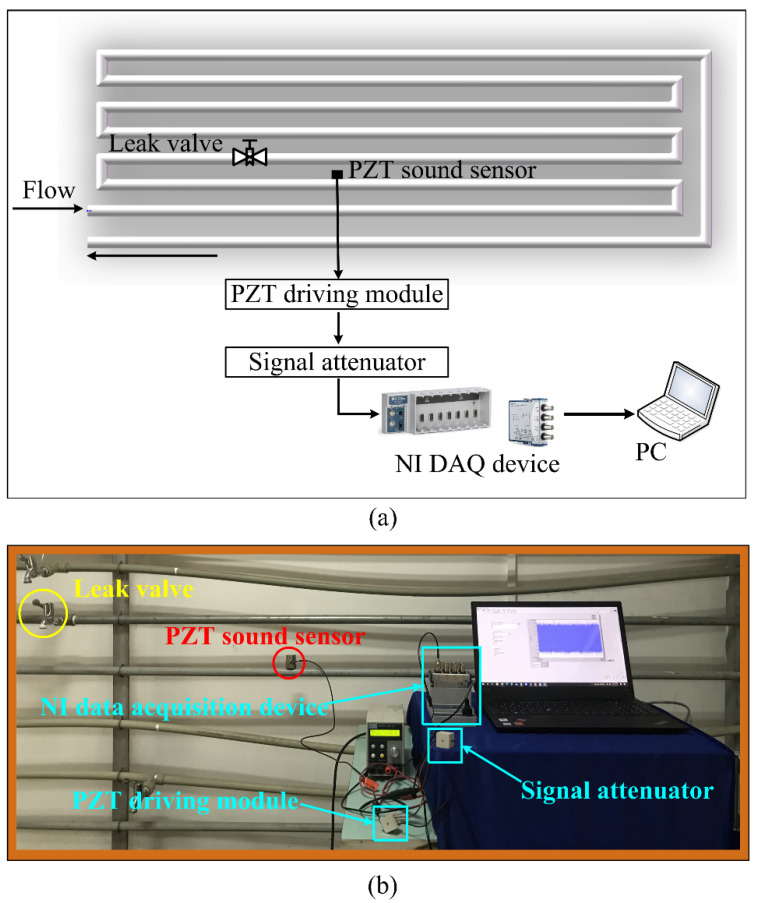
(**a**) Pipe leak test platform diagram. (**b**) The PZT sound sensor, PZT driving module, signal attenuator, NI data acquisition device, and a computer.

**Figure 3:**
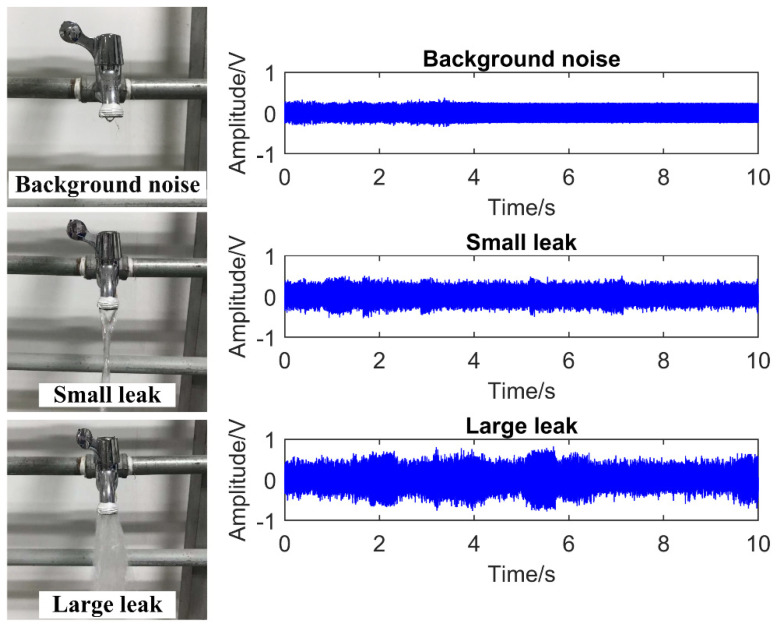
Three leak status of water pipe (**left**), the time−domain waveforms of three leak status (**right**).

To simulate the pipe leak condition, a 200-m water pipeline system was built. Figure 2a shows the pipe leak test platform. The entire platform consists of pipelines, PZT sound sensor, PZT driving module, signal attenuator, National Instruments (NI) data acquisition (DAQ) device, and a computer. The PZT sensor has a resonant frequency of 18 KHz and a frequency range of 0.35–6 KHz; its output voltage signal was amplified by a PZT driving module (preamplifier). To prevent the amplified voltage of the PZT sensor from exceeding the input range of DAQ device, we designed a signal attenuator between the preamplifier and DAQ card. The maximum sampling rate of DAQ card is 1 MHz. Figure 2b shows the PZT sensor was mounted on the pipe away from the leak source. Different opening degrees of the faucet were used to simulate leak conditions. Three leak situations were utilized: background noise (no leak), small leak, and large leak (shown in Figure 3left). We collected 300 sets of data for each leak situation. Finally, 900 sets of data for the three leak situations were obtained. The right of Figure 3 shows a group of time-domain waveforms corresponding to the above three leak situations. It is not difficult to see that the leak time-domain waveform becomes more and more intense with the increasing leak volume. During the data sampling process, the sampling rate was set at 10 KHz, and the single sampling time was 10 s.

### 4.3. Feature Extraction of VAE Data

**Figure 4:**
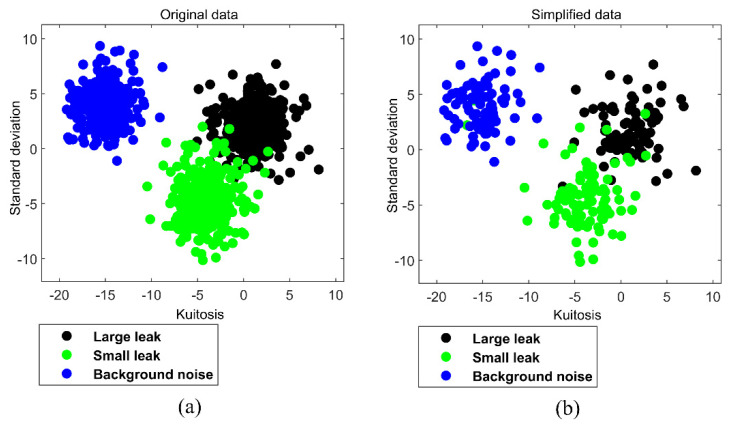
(**a**) Original 2−D pipe leak status samples. (**b**) Simplified 2−D pipe leak status samples.

Eight statistical indices were used to extract feature values from the collected leak VAE data. Subsequently, these feature values were used to construct a training data set *T* for classification. To facilitate the visualization of the classification process, we selected standard deviation and kurtosis feature values as an example and drew a two-dimensional (2−D) scatter diagram of the feature values (Figure 4a). The classification experiment used this 2−D scatter diagram as a study case.

### 4.4. DT Pre-Processing of Data

It is well known that SVM and its improved algorithms are supervised machine learning algorithms, which are mainly suitable for the training and testing of the small-scale sample. Thus, original leak sample data should be simplified. However, some redundant data within the sample points are not helpful for classification. We used the *DT* algorithm to simplify the original leak sample data. The main process is to retain the mainframe of the samples and remove the redundant points located at the center of the sample points. Figure 4 shows the original sample data and the simplified sample points with redundant data removed.

### 4.5. MLT-KSVC Classification for Leak Detection

**Figure 5:**
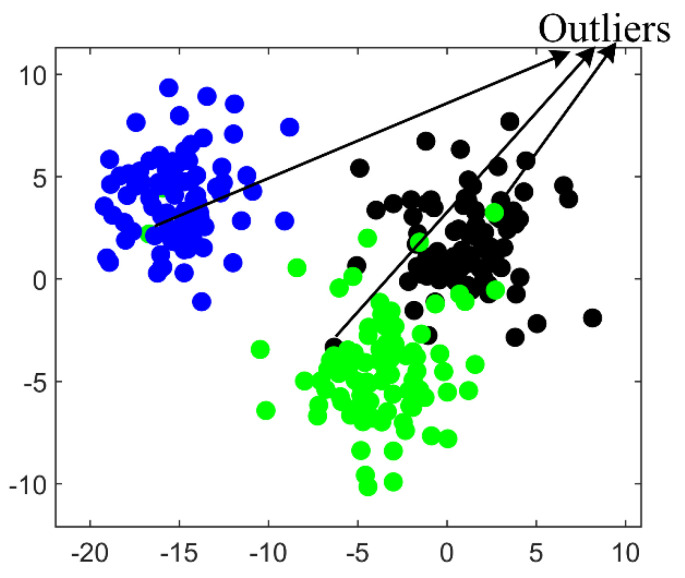
Three typical outliers in leak sample points.

In MLT-KSVC leak classification, the first step was used, MaxEnt frame, to establish the classification weight matrices $W_{1+}$ and $W_{2-}$ for leak samples. In the process of constructing the weight matrix, the data of sample center was given a large weight, while the outliers were given a small weight. Figure 5 shows three typical outliers generated by the interference of environmental noise. Therefore, these sample points can be classified. Here, LST-KSVC was also used to classify the same leak sample points as a comparative experiment example for MLT-KSVC. In the classification process, we used the grid search method to optimize the hyperparameters *C* and *G* of MLT-KSVC and LST-KSVC. In nonlinear MLT-KSVC and nonlinear LST-KSVC classification, we selected the ‘RBF’ as the kernel function, in which all programs were run on MATLAB 2019a.

**Figure 6:**
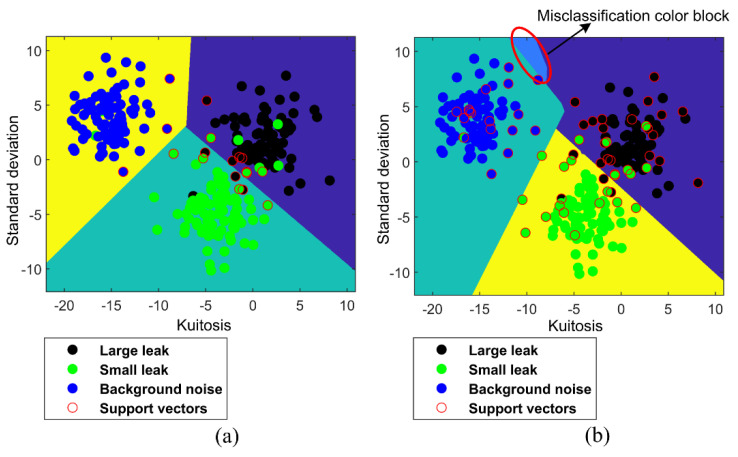
Linear MLT−KSVC and LST−KSVC classifications. (**a**) Linear MLT−KSVC classification for leak samples. (**b**) Linear LST−KSVC classification for leak samples.

Figure 6 shows the classification results of linear MLT-KSVC and linear LST-KSVC, in which black points represent large leaks (+1 class), green points represent small leaks (−1 class), blue points represent background noise (no leak, 0 class), and red circles represent support vector points. It can be seen from Figure 6b that a misclassification color block (within the red oval box) appears in linear LST-KSVC, but linear MLT−KSVC overcomes this shortcoming. By comparing Figure 6a,b, it can be found that the classification color block of linear MLT-KSVC are more regular than linear LST-KSVC, which means that the generalization ability of linear MLT-KSVC is stronger than that of linear LST-KSVC.

**Figure 7:**
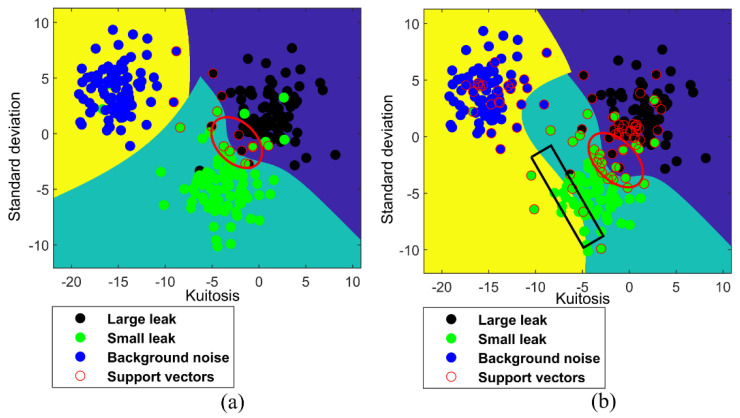
Non-linear MLT−KSVC and LST−KSVC classifications. (**a**) Non−linear MLT-KSVC classification for leak samples. (**b**) Non−linear LST−KSVC classification for leak samples.

Figure 7 shows the classification results of nonlinear MLT-KSVC and nonlinear LST-KSVC algorithms. In an ideal classification state, the boundary inside the red oval box in Figure 7a,b should be similar to a straight line. But the boundary inside the red oval box in Figure 7a,b is not straight, we conjecture that the phenomenon is a negative result caused by outliers. Comparing the red oval box of Figure 7a,b, we found that the nonlinear MLT-KSVC is slightly affected by outliers, but this is much less than the nonlinear LST-KSVC. The classification boundary of nonlinear LST-KSVC is not very regular in Figure 7b. It is not difficult to see that the classification boundary in the black rectangular box is wrong in Figure 7b. We speculate that the misclassification boundary is caused by the overfitting in the nonlinear LST-KSVC. However, the classification boundary (Figure 7a) of nonlinear MLT-KSVC is still more regular compare to that of nonlinear LST-KSVC, which shows that the outliers of sample points have less influence on the linear MLT-KSVC algorithm.

**Figure 8:**
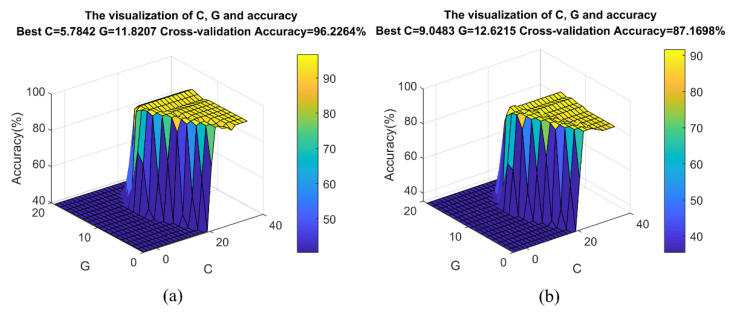
Results of optimal hyperparameter *C*, penalty factor *G*, and cross-validation accuracy. (**a**) MLT-KSVC and (**b**) LST-KSVC.

**Figure 9:**
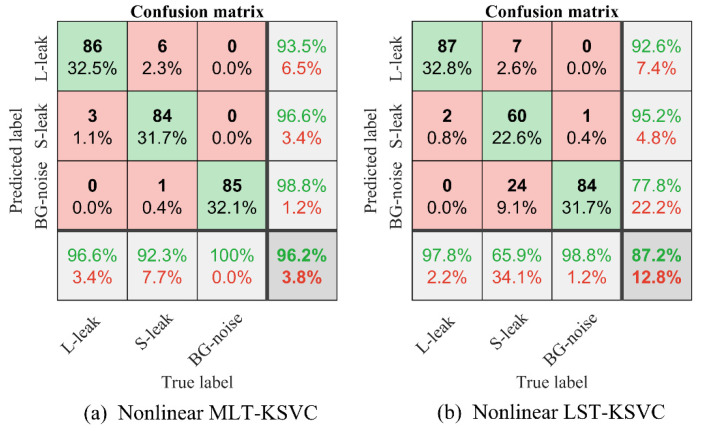
(**a**) The confusion matrix visualization of nonlinear MLT-KSVC. (**b**) The confusion matrix visualization of nonlinear LST-KSVC.

To further compare the performance of the two algorithms, we used MLT-KSVC and LST-KSVC to conduct multiple nonlinear classification experiments to obtain optimal hyperparameter *C*, penalty factor *G*, and cross-validation accuracy based on these sample points. Figure 8 shows a three-dimensional distribution map that combined optimal hyperparameter *C*, penalty factor *G*, and cross-validation accuracy. Ten-fold cross-validation was used to cross-validate the algorithms, which divided the samples into 10 parts, and took 9 parts as training data and 1 part as test data, in turn, to implement experiments. It can be seen from the Figure 8 that the optimal cross-validation accuracy rate of nonlinear MLT-KSVC is as high as 96.2264%, while that of nonlinear LST-KSVC is only 87.1698%. In nonlinear MLT-KSVC classification, the mean of cross-validation accuracy is 96.15%, and the standard deviation of cross-validation accuracy is 0.0330. In nonlinear LST-KSVC classification, the mean of cross-validation accuracy is 87.60%, and the standard deviation of cross-validation accuracy is 0.0610. Then, we can obtain the overall confusion matrix in Table 1 and Figure 9. The following metrics were calculated by us.

(37) $$\mathrm{Accuracy}=\frac{TP+TN+TR}{TP+TN+TR+FP+FN+FR}\cdot 100\%$$

(38) $$\mathrm{Sensitivity}=\frac{TP}{TP+FN+FR}\cdot 100\%$$

In Table 1, TP represents the true positive judgment, TN represents the true negative judgment, TR represents the true rest judgment, FP represents the false positive judgment, FN represents a false negative judgment, and FR represents a false rest judgment. In Figure 9, L-leak represents large leak, S-leak represents small leak, BG-noise represents background noise. In Figure 9a, the overall accuracy of nonlinear MLT-KSVC is 96.2%, and the sensitivity of nonlinear MLT-KSVC is 93.5%. In Figure 9b, the overall accuracy of nonlinear LST-KSVC is 87.2%, and the sensitivity of nonlinear LST-KSVC is 92.6%. 

Then, we changed the feature number of leak samples and used the classical Multi-SVM, TwinSVC, TwinKSVC, LST-KSVC and MLT-KSVC to classify these samples. In these experiments, we still chose ‘RBF’ as the kernel function of the classification algorithm. Table 2 shows the comparison of the classification accuracy and calculation time of the classical Multi-SVM [28], TwinSVC [29], TwinKSVC [30], LST-KSVC and MLT-KSVC. In Table 2, different feature number correspond to different feature types, and the corresponding relationship between them is as follows:

1. Feature number = 1: standard deviation.
2. Feature number = 2: standard deviation, kurtosis.
3. Feature number = 3: standard deviation, kurtosis, variance.
4. Feature number = 4: standard deviation, kurtosis, variance, RMS.
5. Feature number = 5: standard deviation, kurtosis, variance, RMS, margin.
6. Feature number = 6: standard deviation, kurtosis, variance, RMS, margin, mean.
7. Feature number = 7: standard deviation, kurtosis, variance, RMS, margin, mean, waveform factor.
8. Feature number = 8: standard deviation, kurtosis, variance, RMS, margin, mean, waveform factor, peak factor.

From Table 2, it can be seen that the calculation time of nonlinear MLT-KSVC is similar to that of nonlinear LST-KSVC, but the accuracy is higher than that of nonlinear LST-KSVC and other classifiers.

### 4.6. Application Discussion of MLT-KSVC Classification in City Water Pipelines

**Figure 10:**
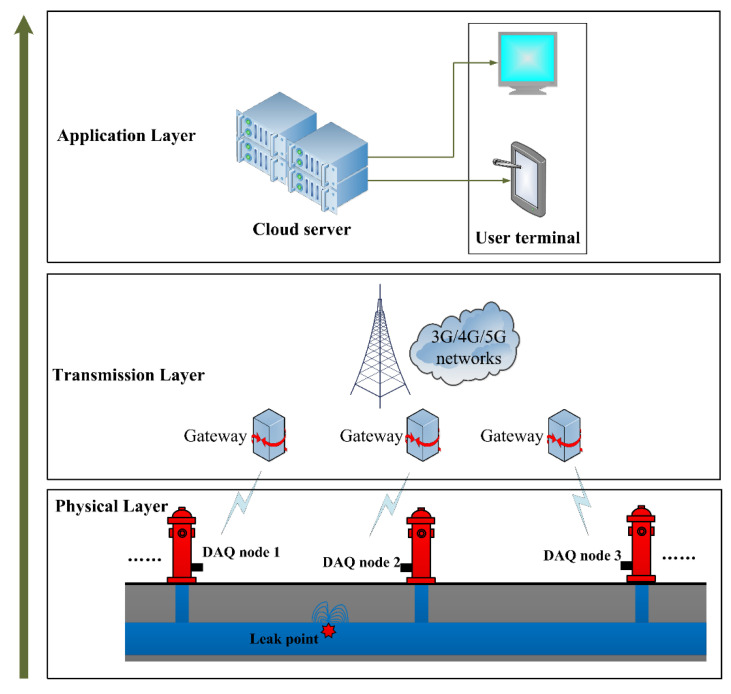
The entire leak recognition system for city water pipeline.

Then, we will briefly discuss the MLT-KSVC application for leak recognition of city water pipelines. Figure 10 presents a complete schematic diagram of pipeline leak recognition system. From bottom to top, the recognition system is divided into three layers.

1. The first is the physical layer, which uses many data acquisition (DAQ) nodes to collect the pipeline acoustic vibration data and transmit the pipe data to the relay gateway. The DAQ node comprises a sensing module, analog to digital conversion (ADC) module and data wireless transmission module. In order to obtain the pipeline position information from the DAQ node, it is necessary to take the DAQ node number as the frame header of the pipe data when transmitting the pipeline data frame.
2. The second is data transmission layer. After the relay gateway received the wireless pipe data frame, and then the relay gateway uses the public networks (3G/4G/5G) to transmit the pipe data to the cloud server.
3. The third is the application layer. In this layer, the cloud server will run the MLT-KSVC classification algorithm model. The application layer performs feature extraction preprocessing on the pipeline data; then, the classification model will complete the leak recognition task based on the extracted features. The user can check the leak recognition results through the terminal device, and take corresponding maintenance and repair plans for different leak statuses.

## 5. Conclusions

This paper used the MaxEnt model to establish two weight matrices that can be used for classification. These weight matrices can make the MLT-KSVC classification algorithm reduce sensitivity to outliers. From the linear classification result, a misclassification color block appears in linear LST-KSVC, but the linear MLT-KSVC can aovid this shortcoming. Whether in linear or nonlinear classification results perspective, the MLT-KSVC has more regular classification color blocks for leak samples than corresponding LST-KSVC, which proves that MLT-KSVC is less sensitive to outliers, and the generalization ability of MLT-KSVC is stronger than that of LST-KSVC. The MLT-KSVC and LST-KSVC took the similar calculation time to complete the classification, but still much less than Multi-SVM, TwinSVC and TwinKSVC. Although the calculation time remains comparable to that of LST-KSVC, MLT-KSVC is more accurate in classifying leak sample points. However, MLT-KSVC has some limitations. For instance, when the data sample is very large, the MLT-KSVC algorithm may crash and even terminate. Solving this problem will be an important research direction in the future.

## Figures and Tables

**Table 1:** Confusion matrix.

Predicted +1	TP	FN	FR
Predicted −1	FP	TN	FR
Predicted 0	FP	FN	TR
	True +1	True −1	True 0

**Table 2:** Leak classification accuracy and calculation time results of Multi-SVM, TwinSVC, TwinKSVC, LST-KSVC and MLT-KSVC.

Feature Number	Outputs	Multi-SVM	TwinSVC	TwinKSVC	LST-KSVC	MLT-KSVC
1	accuracy (%)	62.2641	74.7170	75.8491	85.6604	92.4528
	calculation time (s)	3.0124	2.1105	1.9012	0.2207	0.2310
2	accuracy (%)	60.3773	74.3396	76.9811	87.1698	96.2264
	calculation time (s)	2.6820	2.0164	1.9146	0.2298	0.2304
3	accuracy (%)	60.7547	75.4717	77.7358	87.9245	96.9811
	calculation time (s)	2.7504	2.3738	1.9872	0.2860	0.2834
4	accuracy (%)	61.1321	73.2075	77.3585	89.4340	95.8491
	calculation time (s)	2.7018	2.1083	1.8509	0.3019	0.3095
5	accuracy (%)	63.0189	75.8491	78.1132	90.1887	95.0943
	calculation time (s)	2.7239	2.1834	1.8957	0.3672	0.3932
6	accuracy (%)	63.7736	75.0291	78.4906	90.9434	97.7358
	calculation time (s)	2.6713	2.5263	1.9863	0.4153	0.4035
7	accuracy (%)	63.3962	76.6038	78.8679	93.2075	98.1132
	calculation time (s)	3.1121	2.5967	2.1558	0.4507	0.4518
8	accuracy (%)	64.1509	76.2264	78.8680	93.9623	97.3585
	calculation time (s)	3.2386	2.6093	2.4853	0.4964	0.5086

## Data Availability

The data presented in this study are available on request from the corresponding author.
